# Supplementary material for: Genome-Wide Association Study in Bread Wheat Identifies Genomic Regions Associated with Grain Yield and Quality under Contrasting Water Availability
Source: Int J Mol Sci. 2022 Sep 12;23(18):10575. doi: 10.3390/ijms231810575 (PMC9505613; doi:10.3390/ijms231810575)
Supplement: Supplementary file 1 [file ijms-23-10575-s001.zip › GWAS_Supplementary revision.pdf]

Genome-wide association study in bread wheat identifies genomic regions associated with grain yield and quality under contrasting water availability

Nikolai Govta[1], Iris Polda[2], Hanan Sela[3], Yafit Cohen[4], Diane M. Beckles[5], Abraham Korol[1], Tzion Fahima[1], Yehoshua Saranga[2*], and Tamar Krugman[3]*

[3]Tamar Krugman, Institute of Evolution, University of Haifa, Abba Khoushy Ave 199, Haifa, 3498838, Israel.

[*]Correspondence: Tamar Krugman tkrugman@evo.haifa.ac.il

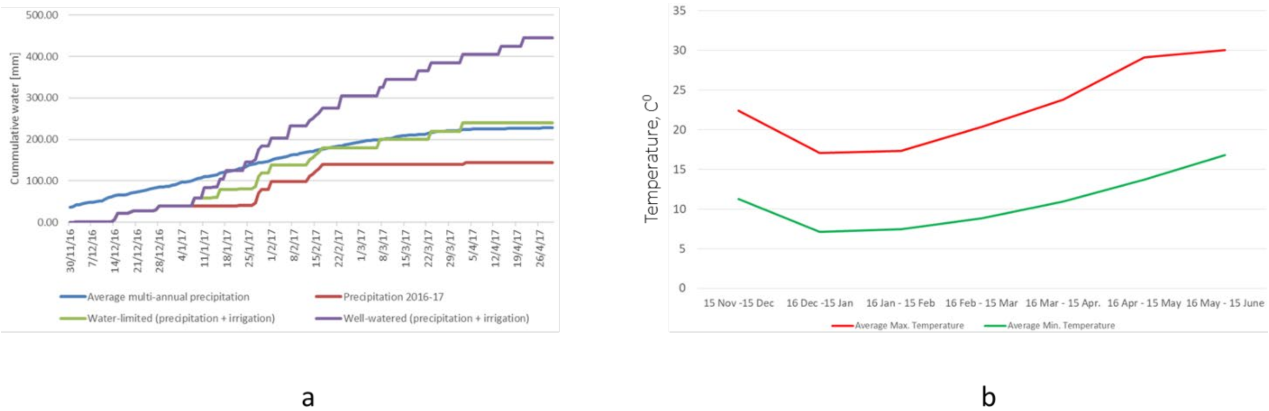

Figure S1. The experiment in Urim 2016-2017 included (a) water status in the two water regimes well-watered and water-limited conditions and (b) temperature regimes.

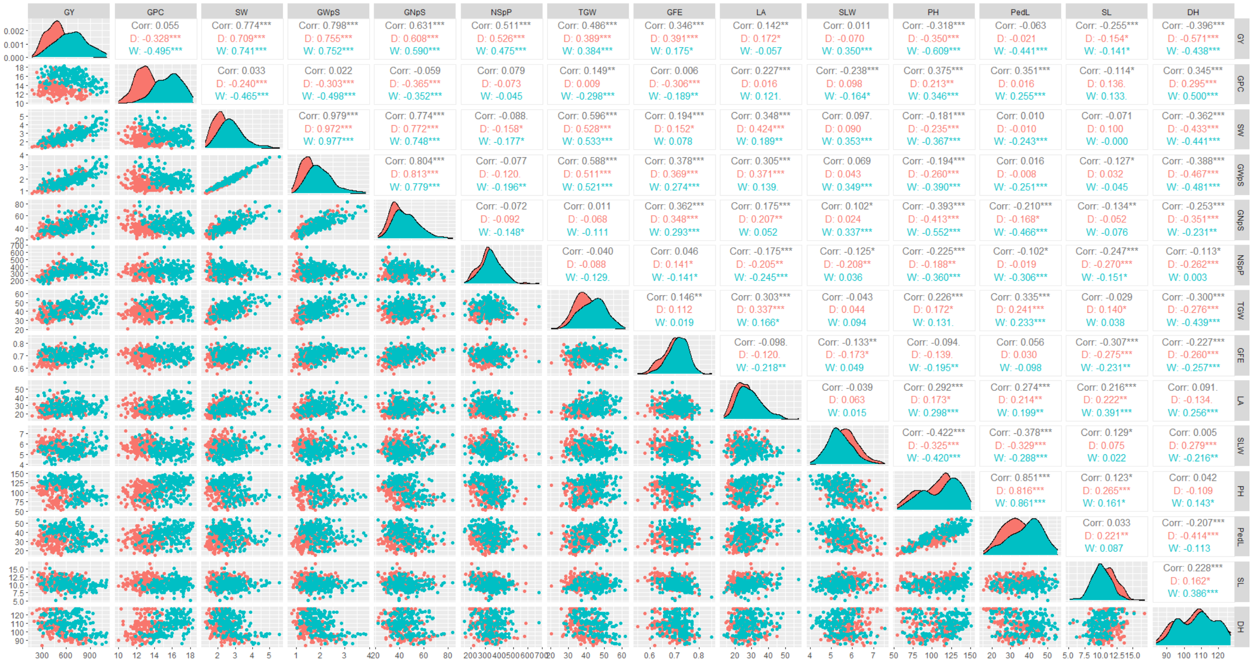

Figure S2. Scatter diagram, correlation matrix and distribution of phenotypic data in 232 wheat accessions under water-limited (D) and well-watered (W) conditions. The top right panel shows the matrix represented includes three correlations - overall, D and W. The left-down panel showed scatter plots between two water regimes. Diagonal is the present distribution of phenotypes. **Key**: grain yield (GY, g/m^2), grain protein content (GPC, %), spike weight (SW, g), grain weight per spike (GWpS, g), grain number per spike (GNpS, #), number of spikes per plot (NSpP, #), thousand grain weight (TGW, g), efficiency of grain filling (GFE, #), leaf area (LA, cm^2), specific leaf weight (SLW, mg/cm^2), plant height including spike (PH, cm), peduncle length (PedL, cm), spike length (SL, cm), days-to-heading (DH, #)

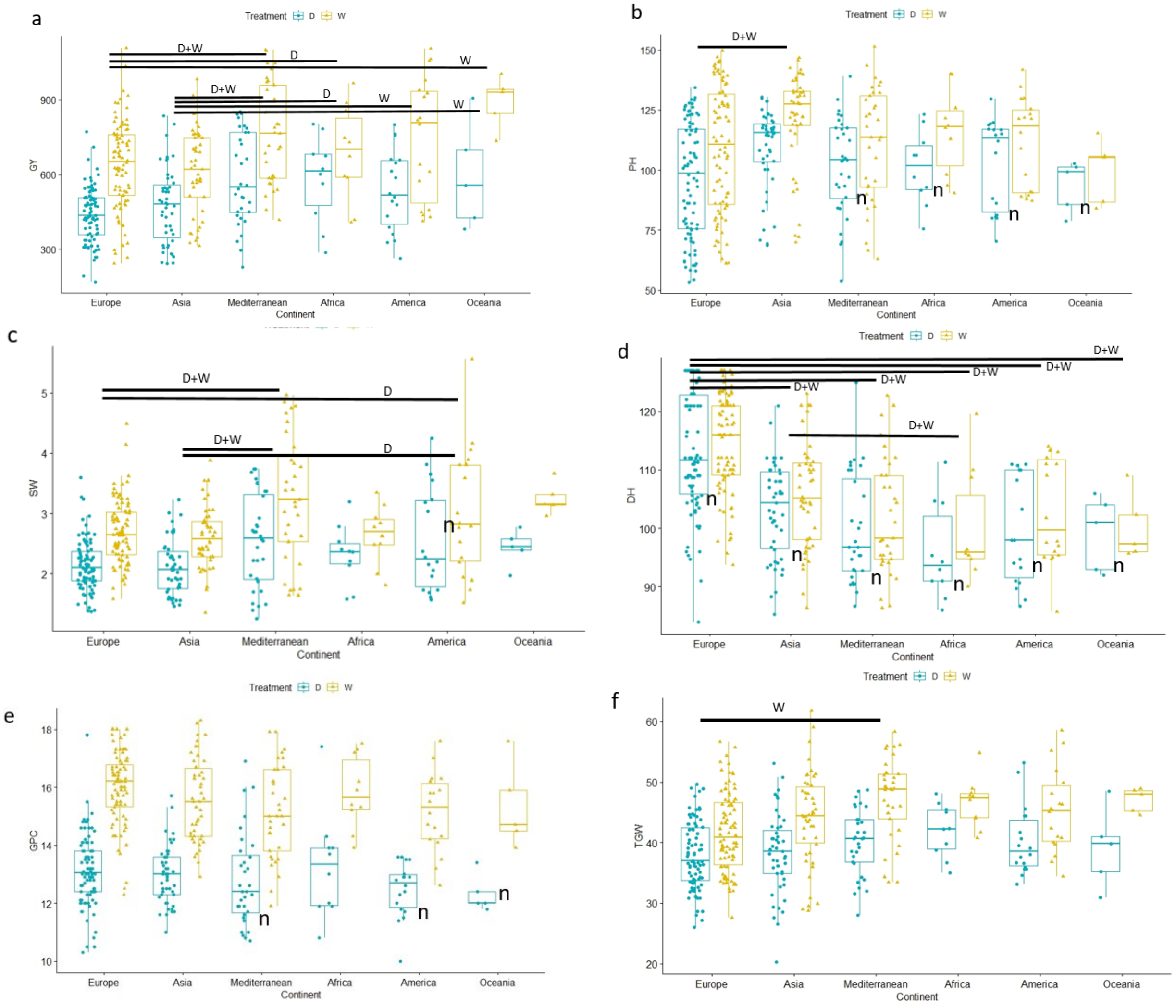

Figure S3. Analysis of phenotypic traits in 232 wheat accessions under water-limited (D) and well-watered (W) conditions by geographic origin (Europe, Asia, the Mediterranean, Africa, America, and Oceania). a – grain yield (GY), b – plant height including spike (PH), c – spike weight (SW), d – days-to-heading (DH), e – grain protein content (GPC), f – thousand grain weight (TGW). The black line shows significant differences between water-limited (D) and well-watered conditions (W) or D+W conditions. n - not significant (between two conditions).

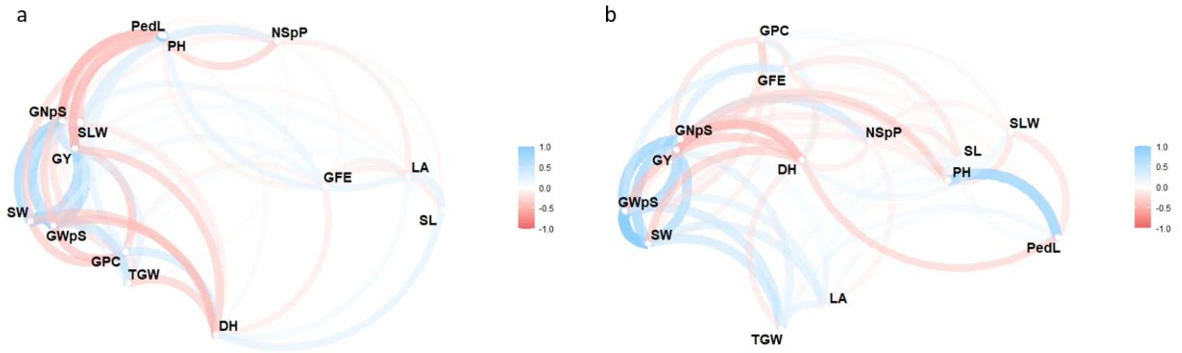

Figure S4. Correlation network PC analysis. Data collected under a) well-watered and b) water-limited conditions. Red line – negative correlation; blue line – positive correlation. Key: grain yield (GY), grain protein content (GPC), spike weight (SW), grain weight per spike (GWpS), grain number per spike (GNpS), number of spikes per plot (NSpP), thousand grain weight (TGW), efficiency of grain filling (GFE), leaf area (LA), specific leaf weight (SLW), plant height including spike (PH), peduncle length (PedL), spike length (SL), days-to-heading (DH).

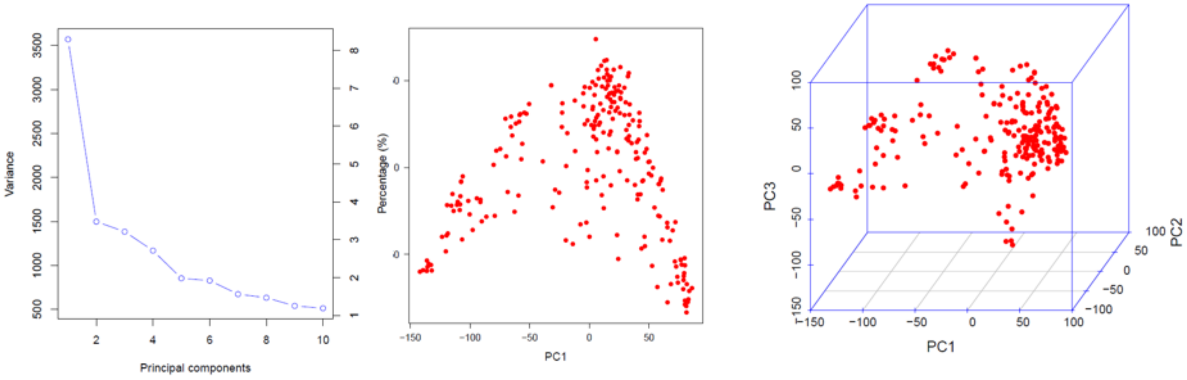

Figure S5. Principal component analysis showing the relatedness of 232 wheat accessions based on 71,571 SNPs.
